# Delimiting Allelic Imbalance of *TYMS* by Allele-Specific Analysis

**DOI:** 10.1097/MD.0000000000001091

**Published:** 2015-07-13

**Authors:** Emilia Balboa-Beltrán, Raquel Cruz, Angel Carracedo, Francisco Barros

**Affiliations:** From the Grupo de Medicina Xenómica (EB-B, RC, AC), CIBERER, Universidad de Santiago de Compostela, Spain; Fundación Pública Galega de Medicina Xenómica (AC, FB), SERGAS, Santiago de Compostela, Spain; and Center of Excellence in Genomic Medicine Research (CEGMR) (AC), King Abdulaziz University, Jeddah, KSA.

## Abstract

Allelic imbalance of *thymidylate synthase (TYMS)* is attributed to polymorphisms in the 5′- and 3′-untranslated region (UTR). These polymorphisms have been related to the risk of suffering different cancers, for example leukemia, breast or gastric cancer, and response to different drugs, among which are methotrexate glutamates, stavudine, and specifically 5-fluorouracil (5-FU), as TYMS is its direct target. A vast literature has been published in relation to 5-FU, even suggesting the sole use of these polymorphisms to effectively manage 5-FU dosage. Estimates of the extent to which these polymorphisms influence in TYMS expression have in the past been based on functional analysis by luciferase assays and quantification of *TYMS* mRNA, but both these studies, as the association studies with cancer risk or with toxicity or response to 5-FU, are very contradictory. Regarding functional assays, the artificial genetic environment created in luciferase assay and the problems derived from quantitative polymerase chain reactions (qPCRs), for example the use of a reference gene, may have distorted the results. To avoid these sources of interference, we have analyzed the allelic imbalance of *TYMS* by allelic-specific analysis in peripheral blood mononuclear cells (PBMCs) from patients.

Allelic imbalance in PBMCs, taken from 40 patients with suspected myeloproliferative haematological diseases, was determined by fluorescent fragment analysis (for the 3′-UTR polymorphism), Sanger sequencing and allelic-specific qPCR in multiplex (for the 5′-UTR polymorphisms).

For neither the 3′- nor the 5′-UTR polymorphisms did the observed allelic imbalance exceed 1.5 fold. None of the *TYMS* polymorphisms is statistically associated with allelic imbalance.

The results acquired allow us to deny the previously established assertion of an influence of 2 to 4 fold of the rs45445694 and rs2853542 polymorphisms in the expression of *TYMS* and narrow its allelic imbalance to 1.5 fold, in our population. These data circumscribe the influence of these polymorphisms in the clinical outcome of 5-FU and question their use for establishing 5-FU dosage, above all when additional genetic factors are not considered.

## INTRODUCTION

Thymidylate synthase (TYMS) is a key protein in cell division since it is the rate-limiting enzyme in “de novo” synthesis of pyrimidines, essential for DNA synthesis.^1^ Literature refers 3 polymorphisms that control its expression. The first discovered a variable tandem repeat sequence (VNTR) (rs45445694) in the 5′-untranslated region (UTR), ranges from 2 to 9 repetitions, being the most frequent alleles of 2 and 3 repeats (2R and 3R).^1,2^ Luciferase assay reported an increment of expression from 2 to 4 fold of the plasmid 3R compared with the plasmid 2R,^3–5^ these data were corroborated by an increased mRNA expression of 3.6 fold in carriers of the 3R allele in a cohort of patients with metastatic colorectal cancer, but not in another with primary colorectal cancer.^1,4^ This contradiction was explained with the discovery of a second polymorphism, a G>C change in the second repeat of the 3R allele (rs2853542) (alteration that also occurs in the first repeat of 2R alleles, but quite rarely),^6^ that is believed to abolish an extra binding site for the stimulatory upstream transcription factor 1 (USF-1).^2^ Luciferase assay showed an increased expression up to 2 to 4 fold of the plasmid 3RG compared to other genotypes.^2,5,7^ Combination of these polymorphisms subdivided patients into groups of high (2R/3RG, 3RC/3RG, and 3RG/3RG) and low expression (2R/2R, 2R/3RC, and 3RC/3RC). The inclusion of the C/G polymorphism in the analysis uncovered the association previously masked^4^ between mRNA expression and the genotypes formerly defined as low and high expression.^8^ The association of the third, a 6 basepair deletion, TS1494del6 (rs34489327) in the 3′-UTR, found to decrease mRNA stability by >50%, is also controversial.^8,9^

These polymorphisms have been related to the risk of suffering different cancers, for example leukemia,^10^ breast^11^ or gastric cancer,^12^ and response and/or toxicity to different drugs, among which are methotrexate,^13,14^ stavudine,^15,16^ and specifically 5-fluorouracil (5-FU),^1,7,8,17–19^ as TYMS is its pharmacological target. This drug is an antimetabolite widely used in different cancers, which has the drawback of showing high interpatient variability in the response and toxicity.^20^ Although “in vitro” TYMS knockdown experiments and gene amplification in cell lines and patients’ samples verify the importance of TYMS in 5-FU resistance and toxicity,^21–24^ association studies about the role of these polymorphisms are contradictory, as well as the functional studies performed.^5,25,26^ Limitations of the techniques on which those data were based on would explain the discrepancy, as the artificial genomic environment created in luciferase assay or the problems derived from the use of a reference gene in quantitative polymerase chain reaction (qPCR) assays. Even so, it is noteworthy that a clinical directed study on rectal cancer patients^27^ that aimed to manage 5-FU treatment based on the VNTR genotype of the patients. The premise of this study, patients with poor prognostic harbor the genotype 3R/3R and 3R/4R, contradicts others similar studies, where the authors relate good response with high expression of the TYMS protein^28,29^ or with the alleles 3R/3R,^18,19^ or with the alleles 2R/3G, 3C/3G, and 3G/3G^17^ or no relation at all.^30,31^

In view of the above discussion, we aim to analyze if the differences previously attributed to these polymorphisms are corroborated by allelic-specific analysis “in vivo”, and contribute to the discussion of these polymorphisms as responsible for the variability in 5-FU efficacy and toxicity.

## MATERIALS AND METHODS

Peripheral blood mononuclear cells (PBMCs) were extracted from the samples of 40 patients referred by suspected myeloproliferative disorders (MPDs) and submitted to the Galician Public Foundation for Genomic Medicine. These patients (aged between 31 to 90 years) are suffered thrombocythemia and/or leukocytosis in most cases. Genetic analysis revealed that 11 of them were *JAK2* positive and all were Philadelphia chromosome negative. PBMCs were used because of the good quality of their mRNA. We analyzed samples from the suspected MPD patients because *TYMS* expression is related to the cell proliferation rate,^32^ and therefore it is predicted lower in normal cells. In addition, the stimulatory transcription factor (USF1), that binds in the repeated sequences of the polymorphic 5′-UTR, is ubiquitous in mammalian cells.^33^ Samples were obtained and used after ethical advice of the local Ethics Committee of the FPGMX in accordance with the declaration of Helsinki. The anonymity of the individuals investigated was preserved corresponding to the rules of data protection of the Galician Health Service. Genotyping for the 5′- and 3′-UTR polymorphisms was performed by DNA sequencing and fluorescent fragment analysis, respectively. cDNA allelic imbalance was performed by Sanger sequencing and qPCR for 5′-UTR polymorphisms and fluorescent fragment analysis for 3′-UTR polymorphisms.

### DNA/mRNA Isolation and Reverse Transcription of mRNA

Germline DNA was extracted from leukocytes of peripheral blood using a magnetic particle-based purification kit (Chemagen, Baesweiler, Germany). mRNA was extracted from PBMCs using the Qiagen RNeasy^®^ Mini Kit (Qiagen^®^, CA), and cDNAs were obtained using SuperScript^®^ reverse transcriptase (Invitrogen^TM^) in accordance with the manufacturer's protocols. cDNA quality was assessed in all samples by measuring Abelson murine leukemia viran oncogene homolog 1 (*ABL1*) expression in accordance with the standardized protocol developed by the Europe Against Cancer program.^34^

### Sequencing Assays

The *TYMS* 5′-UTR was amplified by polymerase chain reaction (PCR) using the forward primer 5′-CGCGCGGAAGGGGTCCTG, and the reverse primer 5′-GGAGGATGTGTTGGATCTGC, with the following program: 5 minutes at 95°C, 35 cycles of 30 seconds at 95°C, 30 seconds at 58°C, 90s at 72 °C, and finally 7 minutes at 72°C. After cleaning up with ExoSAP-IT® (GE Healthcare, NJ), sequencing reactions were run using the same primers and the BigDye® Terminator Kit (Applied Biosystems), and the products were purified using Performa DTR plates (EdgeBio, Gaithersburg, MD) and analyzed on an ABI 3730XL capillary sequencer. For quantification, the cDNA samples were compared with their matching DNA.

### Fluorescent Fragment Analysis

The *TYMS* 3′-UTR variants were identified by fluorescent fragment analysis using previously published PCR primers and conditions and the Peak Scanner v.1.0 software.^35^ For quantification, cDNA samples were compared with their matching DNA.

### qPCR Assays

**FIGURE 1:**
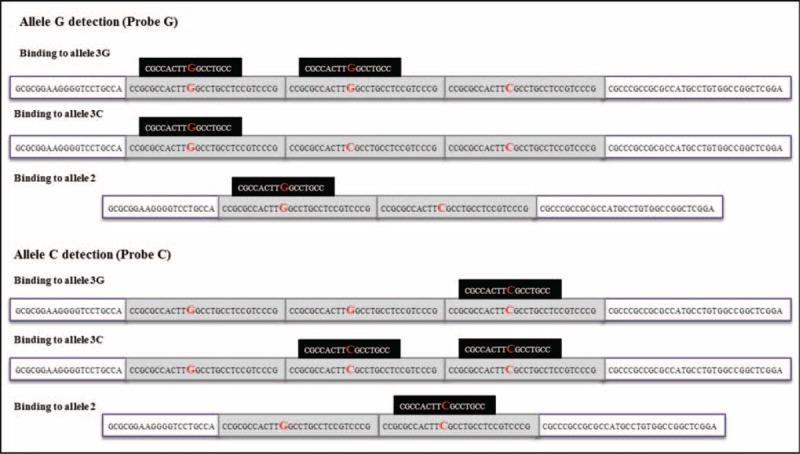
Binding of probes G and C to each 5′-UTR variant. The illustration shows the number of times each probe binds to the sequence depending on the genotype. Gray segments are 28 nucleotide repeats polymorphism, with position 12 emphasized. Probes sequences are represented in black squares, with the variant nucleotide similarly emphasized. 5′-UTR = 5′-untranslated region.

**FIGURE 2:**
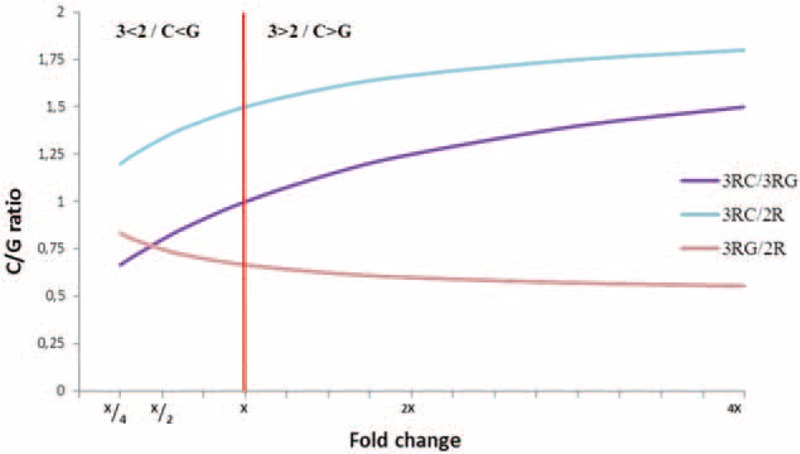
Theoretical C/G ratio of the allele 1 relative to allele 2 for each genotype as function of for the allelic imbalance of x fold. Ratios of C-repeats to G-repeats in cDNA preparations of genotypes A/B (A,B = 2r,3rC,3rG) as functions of the ratio ρ between the amount of A and the amount of B (C-repeat/G-repeat ratio = (n_C,B_ + ρn_C,A_)/(n_G,B_ + ρn_G,A_), where n_C,B_ is the number of C-repeats in allele B, etc.).

Imbalance between *TYMS* alleles differing in their 5′-UTR was quantified by allele-specific qPCR to determine the relative numbers of repeats with C and G in cDNA samples (“C-repeats” and “G-repeats”, respectively); this ratio (“the C/G ratio”) depends on the concentrations of the 2 alleles. The same forward primer used in sequencing and the reverse primer 5′-CTCCGAGCCGGCCACAGG were used to amplify the 5′-UTR *TYMS* region. The hydrolysis probes were: for the C allele 5′-CGCCACTTCGCCTGC with VIC as reported fluorochrome, and for the G allele 5′-CGCCACTTGGCCTGC with 6-Fam as reported fluorochrome, the quencher fluorochrome was MGB for both of them. The G probe binds to 2 repeats on a 3RG allele and only 1 on a 3RC allele, the C probe binds to 2 repeats on 3RC and only 1 on 3RG, and both bind to only 1 repeat on a 2R allele (Figure 1; all 2R alleles were of the type shown in this figure, with a G-repeat followed by the final C-repeat). Measurement of the relative numbers of C- and G-repeats (which requires the binding efficiencies of the probes to be equal but not necessarily 100%) therefore allows the relative concentration of the alleles to be read off from Figure 2, where it is shown the theoretical calculation of the expected values of allelic imbalance for different values of variation for a known genotype.

DNA (300–600 ng) and cDNA (200-300 ng) was amplified in a qPCR assay using 720 nM of each primer and 200 nM of the corresponding hydrolysis probe for the singleplex reaction, or 700 nM of each primer and 125 nM of each hydrolysis probe for the multiplex reaction. qPCR was performed in a StepOnePlus™ System (Applied Biosystems) using TaqMan^®^ Universal PCR Master Mix without Uracil DNA glycosylase (Applied Biosystems) and the following program: 2 minutes at 50°C, 10 seconds at 95°C, 40 × (30 seconds at 95°C, 60 seconds at 65°C). For assay validation, we performed a serial dilution of a homozygous DNA sample (2R/2R); 5 points in a 10-fold dilution were tested in triplicate to calculate the efficiency of the reaction in singleplex. For multiplex assay validation, a single and multiplex assay was performed in parallel in a plate with a serial dilution of a DNA sample with the 3RC/3RG genotype. qPCR efficiency was calculated according to the following equation: E = −1 + 10 [−1/slope].^36^

Expression of each allele was determined by absolute quantification, using a standard curve made of a serial dilution of a DNA with a genotype 3RC/3RG. cDNA measurements under the limit of quantification, calculated according to the Clinical and Laboratory Standards Institute guideline, were excluded for further analysis.^37^ Data reported are in agreement with the Minimum Information for Publication of Quantitative Real-Time PCR Experiments guidelines.^38^

### Statistical Analysis

Statistical analyses were performed using IBM SPSS Statistics 20. Spearman rank correlation was calculated to explore the relationship between variables. One-way nonparametric analyses (Kruskall–Wallis test for multisample data and Wilcoxon test for 2-sample data) were performed to evaluate the existence of differences between groups for quantitative variables. The normalized ins/del values of each sample were analyzed with repeated measures analysis of variance (ANOVA), with DNA/cDNA as within-subjects factor and VNTR as between-subjects factor. A significant *P* value was set as <0.05.

## RESULTS

### Sequencing Analysis

Twenty-six of the 40 DNA samples analyzed were heterozygous for 1 or both 5′-UTR polymorphisms: 9 samples were 3RC/3RG, 8 samples were 2R/3RG, and 9 samples were 2R/3RC.

**FIGURE 3:**
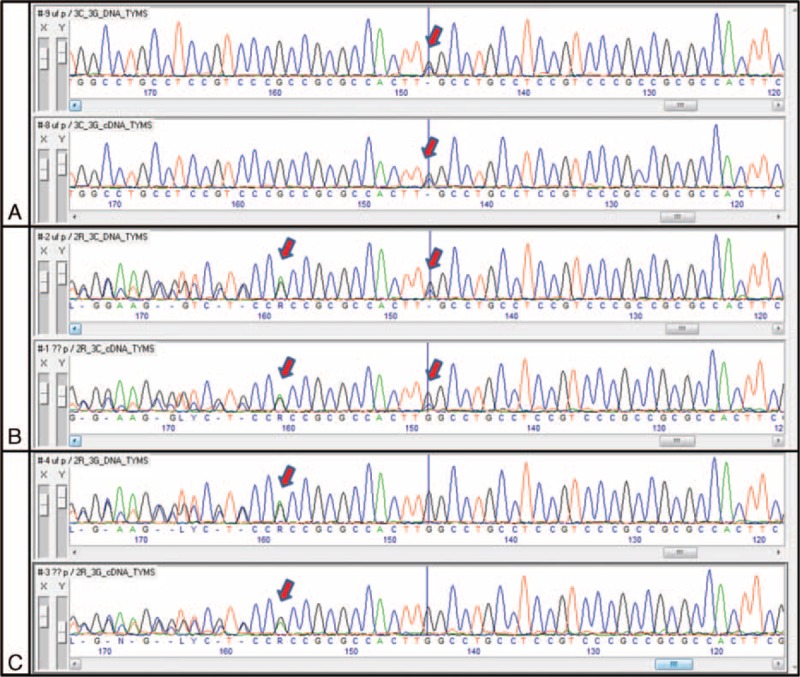
DNA/cDNA comparison of the 5′-UTR *TYMS* polymorphisms in samples with different genotypes: (A) corresponds to sequences from DNA and cDNA samples with 3C/3G genotype; (B) corresponds to sequences from DNA and cDNA samples with 2R/3C genotype; different genotypes; and (C) corresponds to sequences from DNA and cDNA samples with 2R/3G genotype. 5′-UTR = 5′-untranslated region, *TYMS = thymidylate synthase*.

cDNA sequencing was performed in 15 heterozygous 5′-UTR samples: 8 samples were 3RC/3RG, 6 samples were 2R/3RG, and 1 sample was 2R/3RC. DNA/cDNA sample comparison showed an allelic imbalance of <1.5 fold because of either the C/G or the VNTR polymorphisms. The similitude in height of the peaks is shown in the electropherograms (Figure 3).

### qPCR Analysis

We tested allelic imbalance on different genotypes. Replication of the experimental data allowed us to validate the assays, in single and multiplex. qPCR efficiencies data for single and multiplex assays performed in parallel are shown in Table 1.

**TABLE 1:**
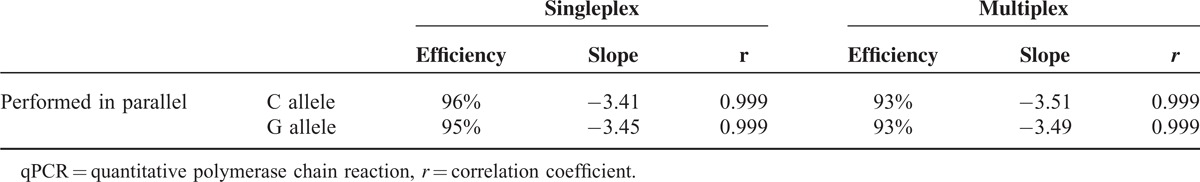
qPCR Efficiency Data Obtained in Single and Multiplex Assay

We analyzed cDNA samples from 19 patients by qPCR assay: 2 of the samples were homozygous (2R/2R), and 17 were heterozygous: 7 samples were 3RC/3RG, 7 were 2R/3RG, and 3 were 2R/3RC. We reanalyzed one of the 2R/2R samples in a second plate, and 3 of the 3RC/3RG samples in 2 more plates, to measure the interplate variation. All cDNAs analyzed were within the quantification cycle range: 24.6–35.

**FIGURE 4:**
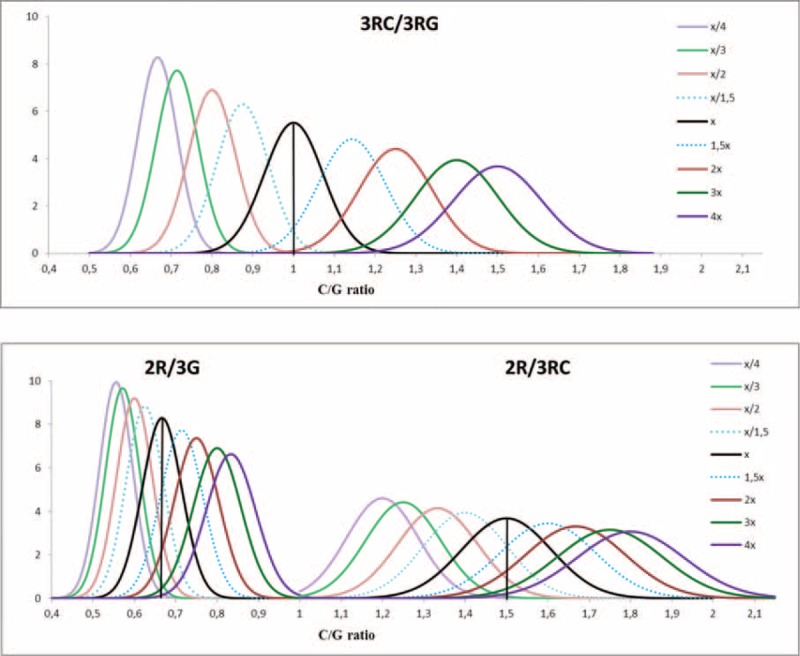
Probability density function calculated according to the median standard deviation of the calibration curve of all plates (σ = 0.072) adjusted by the value of the C/G ratio relative to 1 for the different values of allelic imbalance.

For DNA genotyping, the Spearman correlation between observed and expected values in singleplex assay was 0.89 (*P* < 0.001) and in multiplex assay was 0.87 (*P* < 0.001). Median standard deviation for the replicates in the multiplex for all plates was 0.03 (0.007–0.06). Median standard deviation of the calibration curve of all plates was 0.072. We determine the probability density function for the different C/G ratios of allelic imbalance (Figure 4). Owing to the overlapping of the curves we determine this assay is adequate for measuring differences in allelic imbalance for the 3C/3G genotype but not for the 2/3 genotype.

Nonsignificant differences between plates were found for normalized copy number, DNA_n in the singleplex assay (Kruskall–Wallis test, χ^2^ = 2.45, *P* = 0.29) and in the multiplex assay (χ^2^ = 1.17, *P* = 0.95) and for cDNA_n in the multiplex assay (χ^2^ = 2.3, *P* = 0.80). Lack of plate effect was also shown in the subanalysis of 2 individuals replicated in 3 different plates in the multiplex assay (χ^2^ = 0.85, *P* = 0.65)

### 3C/3G Expression Analysis

Once established the expected values of allelic imbalance of 2 fold is <0.8, when the allele G confers an increase in expression, and >1.25, when the allele C confers an increase in expression (Figure 2). We calculated the expression cDNA values obtained (average 1.09, range: 0.99–1.24) were all within the theoretical range (0.8–1.25) and significantly different from the expected maximum value (1.25) (Z = −2.36, *P* = 0.01). These qPCR data indicate a maximal allelic imbalance of 2 fold of *TYMS* because of the C/G polymorphism.

### Fluorescent Fragment Analysis

**FIGURE 5:**
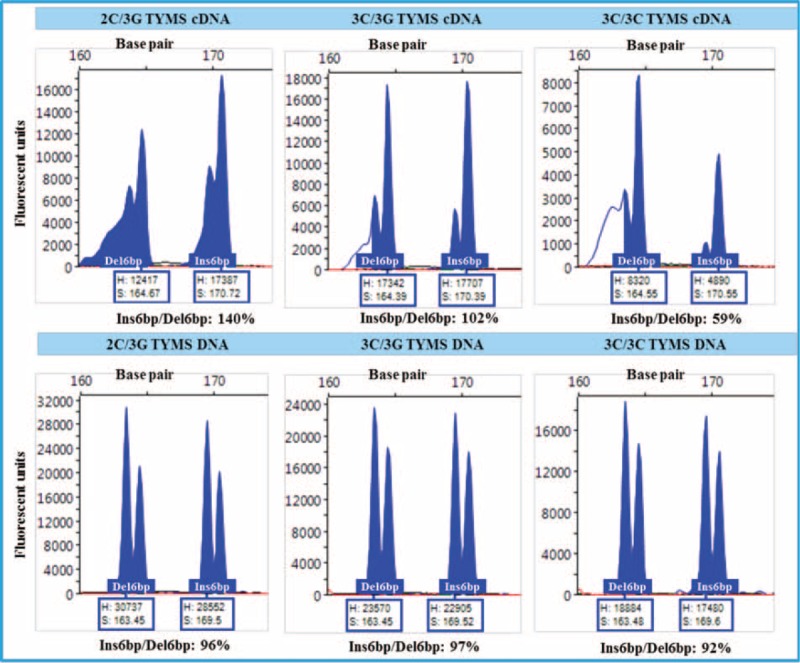
DNA/cDNA comparison of the TS1494del6 polymorphism of TYMS. The top panels correspond to cDNA and the bottom panels to DNA samples. The VNTR genotype of each sample is also indicated. In the panels on the left and on the right are shown the samples with the greatest difference between DNA/cDNA observed in the analyzed group. Each peak is labeled with the height of the peak (H) and size of the fragment in base pairs (S). We are not able to determine the linkage disequilibrium between the 5′-UTR and 3′-UTR polymorphisms but we can establish that the maximal differences between both alleles are of 0.5 fold. TYMS = thymidylate synthase, VNTR = variable tandem repeat sequence, 5′- or 3′-UTR = 5′- or 3′-untranslated region.

**FIGURE 6:**
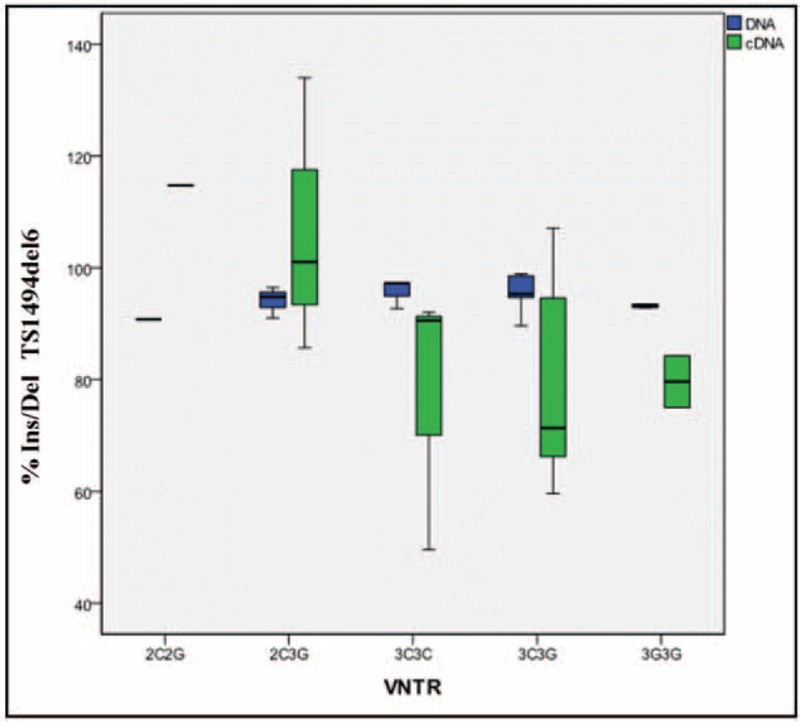
Normalized data of the TS1494del6 polymorphism [ins/del %] of the DNA and cDNA when samples are also segregated by the VNTR polymorphism. DNA data is indicated in blue boxes and cDNA data is indicated in green boxes. None of the samples surpass a 0.5-fold allelic imbalance. VNTR = variable tandem repeat sequence.

We analyzed DNA/cDNA samples of 15 patients heterozygous for the TS1494del6 polymorphism (10 of them also heterozygous for the 5′-UTR polymorphisms). Statistical analysis of the experimental data indicates there are nonsignificant differences in cDNA (Kruskal–Wallis test, χ^2^ = 4.78, *P* = 0.443) because of this genotype. DNA analysis of the same samples allowed us to validate the assay (χ^2^ = 7.49, *P* = 0.187). Moreover, to check the possible influence of the VNTR in the comparison of normalized ins/del values, we performed a repeated measure ANOVA with VNTR as between-subjects factor and the DNA/cDNA as within-subject factor. Nonsignificant effect was found for DNA–cDNA (F = 0.124, *P* = 0.733) or the interaction with the VNTR genotype (*F* = 1.87, *P* = 0.199). Combination of the polymorphisms VNTR and TS1494del6 does not explain the slight difference in expression observed (Figures 5 and 6).

These data indicated a *TYMS* differential expression due to the TS1494del6 polymorphism of <1.5 fold, which also allow narrowing the difference in *TYMS* expression because of the 5′-UTR polymorphisms.

In conclusion, the allelic imbalance in 21 heterozygous samples: 15 with regard to the 3′-UTR polymorphism, and 15 (6 samples were 2R/3RG, 1 sample 2R/3RC, and 8 3RC/3RG heterozygotes) with regard to the 5′-UTR polymorphisms by sequencing analysis and/or qPCR and/or fluorescent fragment analysis, is <1.5 fold.

## DISCUSSION

Contradictory literature on the role of *TYMS* polymorphisms as responsible of variations in TYMS expression led us to reevaluate this issue. We found none of the polymorphisms analyzed were statistically associated with expression. The maximum allelic imbalance of 1.5 determined in the analysis, refutes the previously reported change of 2-4 fold in TYMS expression due to the VNTR polymorphism. For our knowledge, this is the first article quantifying the expression of *TYMS* by allele-specific qPCR, Sanger sequencing, and fluorescent fragment analysis “in vivo.” We are aware that the number of samples is a limitation in the statistical analysis, but this study is not intended to establish a hypothesis but to contrast a previous one. It can be noted that, besides the published fact that TYMS protein expression and activity are related to the cell proliferation rate in human cancer cell lines,^32^ the level of expression of TYMS in PBMCs is high enough to be measured with reasonable precision using qPCR. Furthermore, as the transcription factors that bind in the repeated sequences of the polymorphic 5′-UTR is ubiquitous in mammalian cells,^33^ although the expression of this transcription factor increases or decreases in cancer cells compared with normal cells, and therefore the total expression of *TYMS* changes, the fold change of 1 allele relative to the other is not affected, and the relative expression must be constant.

Some limitations of the previous approaches used would explain the discrepancy.

1. Luciferase assay isolates regions of the gene promoter from its natural environment which prevents the evaluation of *cis*- and *trans*-regulatory elements and the response to “in vivo” microenvironmental stimuli.^39–42^ Thus, data differ depending on the length of the 5′-UTR included in the plasmid.^5^
2. Three of the 6 functional assays were performed in HeLa cells.^5^ These cells have a significantly different expression pattern compared with normal cells besides of high levels of aneuploidy, aberrant chromosomes, and chromothripsis, distinguished by a random pattern of chromosomal shattering.^43^
3. Cell culture conditions affect gene expression, as results from colorectal cell lines grown in a 2D versus 3D cell culture model show.^42^
4. Variations in transcription factors and nucleosome position can alter the results observed “in vitro” versus “in vivo.”^41^
5. Regarding qPCR analysis, one of the major problems is the choice of a reference gene. Several papers used Glyceraldehyde-3-phosphate dehydrogenase (GAPDH) and β-actin^4,9,44–46^; however, GAPDH expression in normal colon epithelium showed a variability up to 2x between samples and β-actin is not suitable for qPCR assay.^47,48^ Both genes have pseudogenes that can interfere with the assay.^48^

By contrast, the only variables in qPCR allele-specific are the tested polymorphisms and the ones in linkage of disequilibrium. Allele-specific qPCR analysis avoids the problems derived from the use of a reference gene.^48^ Multiplex qPCR decreases differences in PCR efficiencies both at cDNA level as in qPCR, as well as variations in mRNA stability, quality, and integrity or sample handling, reducing intra- and intersample variability. And, although not traditionally used for gene expression quantification, the accuracy of Sanger sequencing is confirmed by different studies.^49,50^

Narrowing the allelic imbalance of *TYMS* to 1.5 fold brings up the question whether this difference is enough to manage the treatment. Our data are in agreement with a review article that calculated the pharmacogenetic window for the allelic imbalance of *TYMS* in 1.5 to 2.5 fold. This value refutes the thesis of a reliable marker because the pharmacogenetic window set for expression is 5 to 10 fold and increases to 10 to 30 fold for genetic mutations.^25^

In relation to 5-FU, lack of association between these polymorphisms and sensitivity to 5-FU “in vitro” assays supports these data.^51,52^ Meta-analysis performed in colorectal cancer indicates not only a small relative risk in relation to efficacy and toxicity for the allele 2R and insertion 6 base pair, but also a possible publication bias and heterogeneity in the literature reviewed analyzing the 5′-UTR polymorphisms.^53,54^ Combination analysis of the 3 polymorphisms did not increase the odds of toxicity.^54^ These analyses support our data and question the use of these polymorphisms for establishing 5-FU dosage, above all when additional genetic factors are not considered. In this regard, notice a recently published paper indicating the association of a polymorphism in the *Enolase superfamily member 1*, an antisense RNA to *TYMS*, and denying the implication of the previously mentioned polymorphisms in *TYMS*.^55^

The results acquired, even in a low number of samples, allow us to deny the previously established assertion of an influence of 2-4 fold of the rs45445694 and rs2853542 polymorphisms in the expression of *TYMS* and narrow the allelic imbalance of *TYMS*, in our population, to 1.5 fold.
